# Moderate static magnetic field promotes fracture healing and regulates iron metabolism in mice

**DOI:** 10.1186/s12938-023-01170-3

**Published:** 2023-11-15

**Authors:** Shenghang Wang, Yuetong Liu, Chenge Lou, Chao Cai, Weihao Ren, Junyu Liu, Ming Gong, Peng Shang, Hao Zhang

**Affiliations:** 1 Department of Spine Surgery, People’s Hospital of Longhua, Affiliated Hospital of Southern Medical University, No.38 Jinglong Construction Road, Shenzhen, China; 2 School of Life Sciences, Northwestern Polytechnical University, Xi’an, China; 3 Research & Development Institute of Northwestern Polytechnical University in Shenzhen, No. 45, Gaoxin South 9th Road, Nanshan District, Shenzhen, 518057 China

**Keywords:** Moderate static magnetic field, Fracture healing, Bone metabolism, Iron metabolism

## Abstract

**Background:**

Fractures are the most common orthopedic diseases. It is known that static magnetic fields (SMFs) can contribute to the maintenance of bone health. However, the effect and mechanism of SMFs on fracture is still unclear. This study is aim to investigate the effect of moderate static magnetic fields (MMFs) on bone structure and metabolism during fracture healing.

**Methods:**

Eight-week-old male C57BL/6J mice were subjected to a unilateral open transverse tibial fracture, and following treatment under geomagnetic field (GMF) or MMF. The micro-computed tomography (Micro-CT) and three-point bending were employed to evaluate the microarchitecture and mechanical properties. Endochondral ossification and bone remodeling were evaluated by bone histomorphometric and serum biochemical assay. In addition, the atomic absorption spectroscopy and ELISA were utilized to examine the influence of MMF exposure on iron metabolism in mice.

**Results:**

MMF exposure increased bone mineral density (BMD), bone volume per tissue volume (BV/TV), mechanical properties, and proportion of mineralized bone matrix of the callus during fracture healing. MMF exposure reduced the proportion of cartilage in the callus area during fracture healing. Meanwhile, MMF exposure increased the number of osteoblasts in callus on the 14th day, and reduced the number of osteoclasts on the 28th day of fracture healing. Furthermore, MMF exposure increased PINP and OCN levels, and reduced the TRAP-5b and β-CTX levels in serum. It was also observed that MMF exposure reduced the iron content in the liver and callus, as well as serum ferritin levels while elevating the serum hepcidin concentration.

**Conclusions:**

MMF exposure could accelerate fracture healing via promote the endochondral ossification and bone formation while regulating systemic iron metabolism during fracture healing. This study suggests that MMF may have the potential to become a form of physical therapy for fractures.

## Background

Fractures are the most important orthopedic diseases. Epidemiological investigation showed that more than 170 million new cases of fracture in 2019, with this annual figure continuing to rise [1, 2].

Bone fractures were seriously impeding the daily activities of patients and exerting a substantial economic burden. Consequently, the bone fractures have been recognized as a global public health issue. The process of bone metabolism during fracture healing encompasses various stages, including the formation of cartilage callus, the transformation from cartilage callus to a hard callus, and bone remodeling [3, 4]. Enhancing the rate of bone metabolism during fracture healing is a pivotal approach to expedite the recovery process associated with fractures.

Electromagnetic fields have served as a valuable modality in fracture treatment for more than 70 years [5]. In the present, dynamic magnetic fields, notably pulsed electromagnetic fields (PEMFs), have exhibited remarkable effectiveness in the management of fractures, delayed unions, and nonunions [6–8]. Compared with dynamic magnetic fields, the static magnetic fields (SMFs) exert no energy deposition into tissues, and without the risk of thermal and electrical damage. However, the understanding of the effects and mechanisms of SMFs on fracture remains relatively limited.

According to magnetic flux density, SMFs could be divided into hypomagnetic field (HyMF, < 5 µT), weak static magnetic fields (< 1 mT), moderate static magnetic fields (MMFs, 1 mT–1 T), and high static magnetic fields (HiSMFs) [9]. Studies have shown that SMFs could regulate the physiological functions of bone tissue cells, such as osteoblast and osteoclast [10, 11]. MMF can be provided by permanent magnetic materials, such as samarium–cobalt and neodymium, which allow wide applicability at an affordable prize.

Bruce GK et al. found that 0.22 T–0.26 T SMF exposure could enhance the mechanical properties of fracture site in rabbits [12]. In addition, Edela et al. observed that implanting magnetic devices of mutual attraction at the fracture exit could promote fracture healing in rats [13]. Nuri et al. found that intramedullary implants incorporating SMFs accelerated the fracture healing in rabbit femur [14]. Our previous studies showed that the external exposure to MMF could alleviate bone loss in mice [15, 16]. Asteinza Castro IM et al. found that 35 mT SMF could augment the effects of photobiotherapy in promoting fracture healing in dog [17]. Nonetheless, the precise impact of MMFs on fracture healing and the state of bone metabolism during this process remain an area of uncertainty.

The impact on metal ion transport and metabolism is one of the biological mechanisms of SMFs [18]. The balance of iron metabolism is closely related to bone health, and abnormal iron metabolism may cause diseases such as osteoporosis and increase the probability of fractures [19]. Different magnetic field environments can affect bone state by regulating the systemic and cellular metabolism [20, 21]. However, the effect of MMFs exposure on iron metabolism in vivo during fracture healing remains unclear.

In this study, we examined the changes of histomorphology, mechanical properties and serum bone metabolism indexes at various stages of the fracture healing process. Aim to elucidate the impact of MMFs whole body exposure on fracture healing and the associated bone metabolism mechanisms. Meanwhile, the iron content in liver and fracture callus, and serum ferritin and hepcidin level have been detected to evaluate the effects of MMF exposure on iron metabolism during fracture healing.

## Results

### Construction of moderate static magnetic field exposure system

**Fig. 1:**
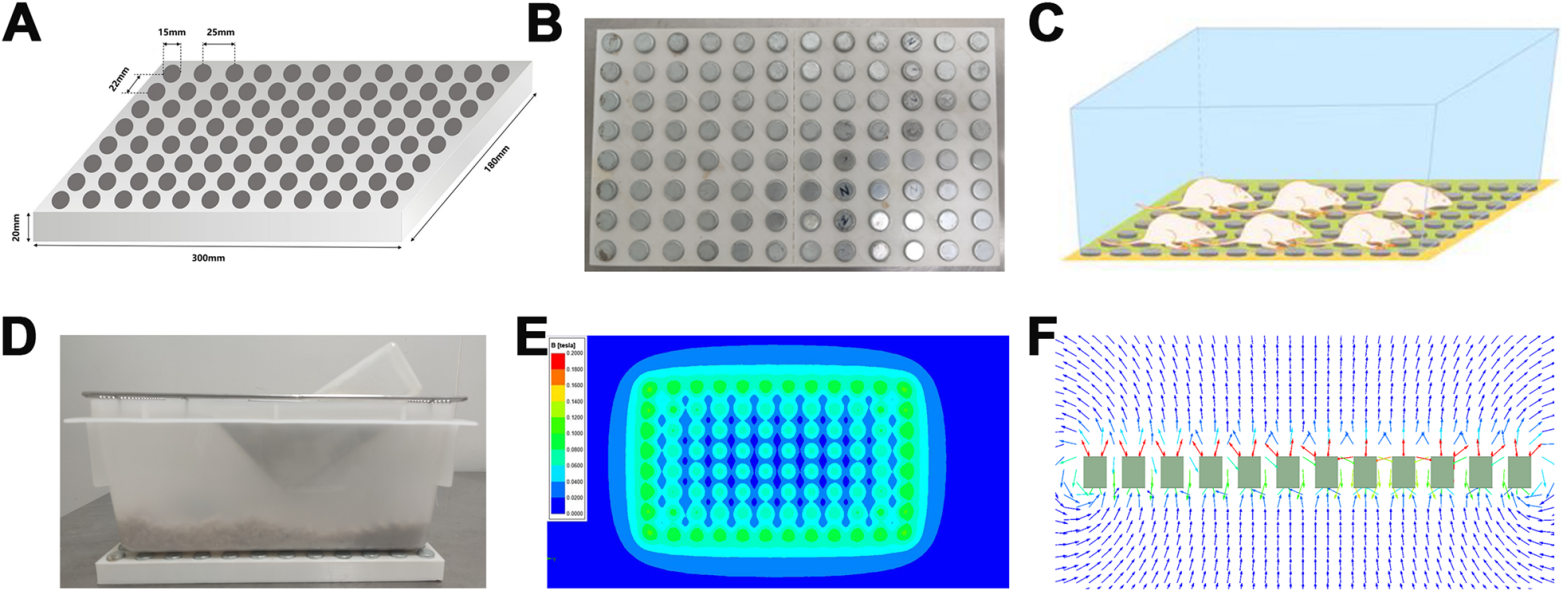
Moderate static magnetic field (MMF) exposure system for mice. **A**, **B** Diagram of magnetic plates that provide MMF. **C**, **D** Relative position of mice during exposure to MMF. **E** Magnetic field distribution in the mouse exposure area, 1 cm above the magnetic plates. **F** Direction and distribution of magnetic induction lines in magnetic exposure systems

The moderate static magnetic field (MMF) environment was generated by 96 neodymium N42 permanent magnets, and each magnet with a diameter of 15 mm and a height of 20 mm (Fig. 1A, B). During this study, the mice were feeding in normal cages, and the cages was placed above the magnetic plates (Fig. 1C, D). The cages are constructed from Polypropylene (PP) material, ensuring they do not disrupt the distribution of the static magnetic field. In control group, the mice breeding cages were was also placed above the plates without real magnets. The simulation results obtained using ANSYS software (ANSYS, Inc., Canonsburg, USA) revealed that the magnetic field intensity within the fracture site of mice subjected to MMF exposure ranged from 0.05 T to 0.5 T (1 cm above the magnetic plate) (Fig. 1E). The magnetic field direction and distribution of the magnetic plates was N pole facing up, as shown in Fig. 1F.

### Effects of MMF exposure on the blood in mice

In this study, the effects of the MMF exposure on routine blood parameters were evaluated by the white blood cell count (WBC), lymphocyte count (LYMPH), red blood cell count (RBC), hemoglobin (HGB), mean corpuscular hemoglobin (MCH) and platelet count (PLT). The results showed that MMF exposure reduced the WBC on day 14 postfracture. However, there were no significant changes observed in LYMPH, RBC, HGB, MCH, and PLT. Meanwhile, the blood parameters in mice subjected to 28 days of MMF exposure were not significantly different from those in control animals (Table 1).

**Table 1:** Effects of MMF exposure on routine blood parameters in the fractured mice

	Blood parameter	Ctrl	MMF
Day 14	WBC (10^9^/L)	4.47 ± 0.61	3.78 ± 0.42*
	LYMPH (10^9^/L)	3.27 ± 0.86	2.70 ± 0.66
	RBC (10^12^/L)	9.70 ± 0.696	10.15 ± 0.53
	HGB (g/L)	133.83 ± 10.94	142.33 ± 7.42
	MCH (pg)	13.82 ± 0.18	14.02 ± 0.12
	PLT (10^9^/L)	812.67 ± 110.11	769.33 ± 77.73
Day 28	WBC (10^9^/L)	4.17 ± 0.93	3.92 ± 1.12
	LYMPH (10^9^/L)	3.44 ± 0.89	2.73 ± 0.69
	RBC (10^12^/L)	9.48 ± 0.49	9.33 ± 0.73
	HGB (g/L)	130.33 ± 5.54	135.50 ± 7.45
	MCH (pg)	13.73 ± 0.26	13.87 ± 0.47
	PLT (10^9^/L)	818.66 ± 145.61	801.67 ± 90.87

Values are means ± SD

*WBC*  white blood cell count; *LYMPH* lymphocyte count; *RBC* red blood cell count; *MCV* mean corpuscular volume; *HGB* hemoglobin; *MCH* mean corpuscular hemoglobin; *PLT* platelet count

*P < 0.05 (*n* = 8)

### Effects of MMF exposure on the microstructure of fracture callus in mice

**Fig. 2:**
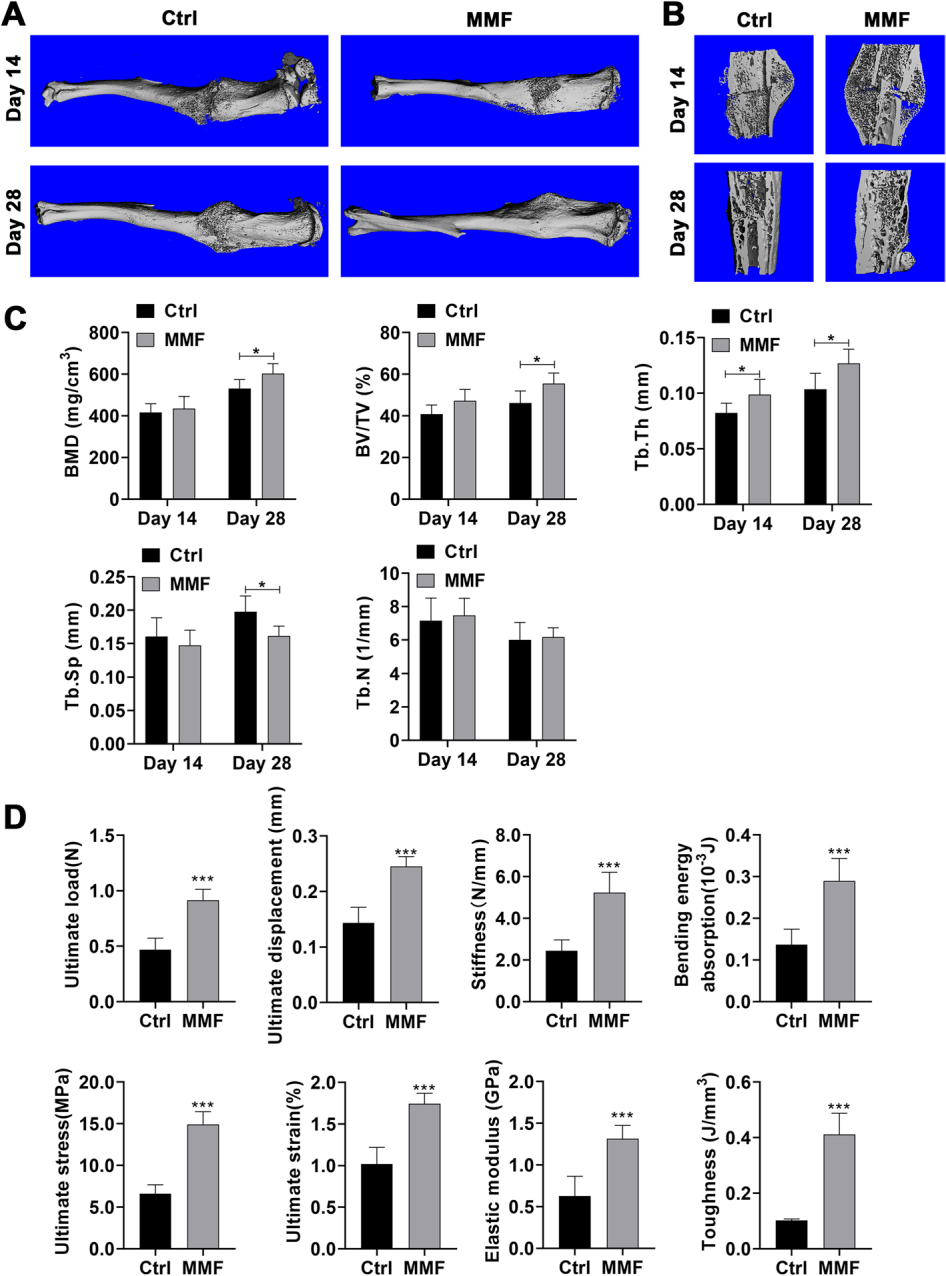
Effects of MMF exposure on microstructure and of biomechanical properties of callus during fracture healing. **A** Representative 3 D dimensional images from Micro-CT scanning in fractured tibia.** B** Representative longitudinal sections of 3D-dimensional reconstructed calluses. **C** Quantitative analysis of structural parameters of fracture calluses, including bone mineral density (BMD), bone volume fraction (BV/TV), trabecular thickness (Tb.Th), trabecular number (Tb.N) and trabecular separation (Tb.Sp), in fracture calluses. **D** Structural mechanics properties, including ultimate load, ultimate displacement, stiffness, and bending energy absorption, and material properties, including ultimate stress, ultimate strain, elastic modulus, and toughness were calculated via the three-point bending test. *n* = 8. Data represent the mean ± SD. *p < 0.05, ***p < 0.001

Representative reconstructions of fractured tibia and callus from 3D micro-CT data sets are shown in Fig. 2A, B. The axial longitudinal section shows that significant the fracture line can still be observed in the MMF group and the control group on day 14 postfracture, and there are a large number of new woven bones basically bridging at the callus. On day 28 postfracture, neither the control group nor the MMF group exhibited any cortical bone gaps within the callus.

Statistical analysis demonstrated that, the callus bone mineral density (BMD), and callus mineralized volume fraction (BV/TV, %) at fracture sites were significantly increased by MMF exposure on day 28 postfracture (Fig. 2C). Meanwhile, the mice in MMF exposure group exhibited significantly higher trabecular thickness (Tb.Th) and lower trabecular separation (Tb.Sp) on the 28th day postfracture. While, MMF exposure does not cause significant changes in Tb.N during fracture healing (Fig. 2C).

### Effects of MMF exposure on mechanical properties of fracture callus in mice

The influence of 28 days of MMF exposure on the mechanical properties of fractured tibia was evaluated via three-point bending test and shown in Fig. 2D. The results showed that MMF exposure significantly improved the structural parameters of the fractured tibia, including ultimate load, ultimate displacement, stiffness, and bending energy absorption. Meanwhile, the material properties of fractured tibia within the MMF group including ultimate stress, ultimate strain, elasticity modulus, and toughness exhibited significantly increases in comparison with the control group.

### Effects of MMF exposure on cartilage and mineralized bone matrix of fracture callus in mice

**Fig. 3:**
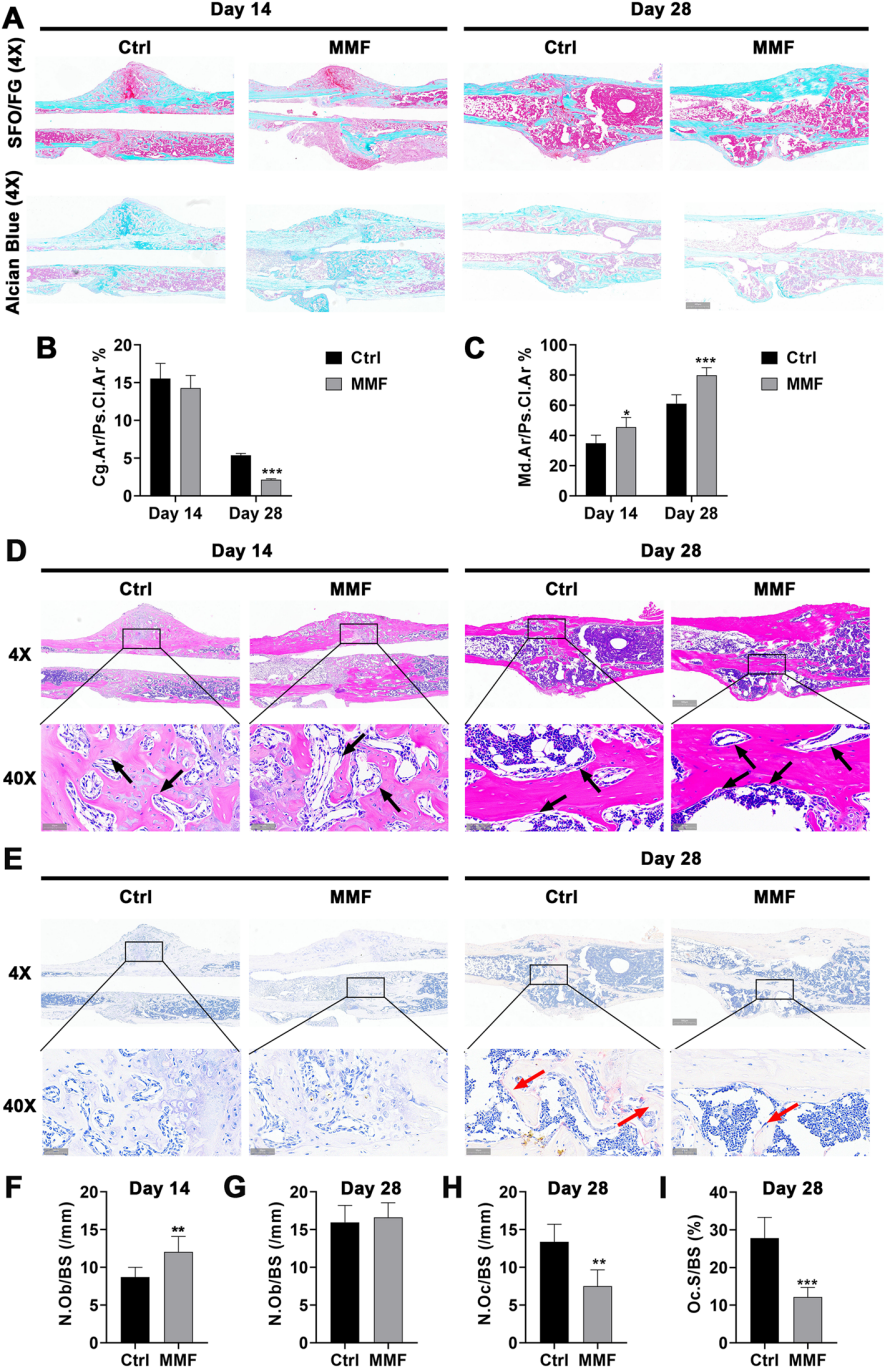
Effects of MMF exposure on the ratio of cartilage and mineralized bone matrix, and the distribution of osteoblasts and osteoclasts on the surface of bone trabeculae in callus. **A** Representative images of SFO/FG and Alcian blue staining on paraffin sections, the cartilage area (SFO and Alcian blue staining positive area) and mineralized area (FG staining positive area) of fracture callus were shown in the figure. **B**, **C** Histomorphometric quantification of cartilage area versus periosteal callus area (Cg.Ar./Ps.Cl.Ar.%) (**B**) and mineralized area versus periosteal callus area (Md.Ar./Ps.Cl.Ar.%) (**C**) between control and MMF exposure group during the fracture healing stage. **D** H&E staining of fracture callus; **E** TRAP staining of fracture callus; **F** osteoblast (black arrow point in **A**) number per bone surface (N.Ob/BS) on day 14 postfracture; **G** N.Ob/BS on day 28 postfracture; **H** osteoclast (red arrow point in **B**) number per bone surface (N.Oc/BS) on day 28 postfracture; **I** osteoclast surface per bone surface (Oc.S/BS) on day 28 postfracture. Scale bar = 100 μm in original images (4X), and Scale bar = 50 μm in enlarged images (40X). *n* = 8. Data represent the mean ± SD. **p < 0.01, ***p < 0.001

The Safranin O and Alcian blue were used to detect the distribution of cartilage tissue on paraffin sections, which could be stained with two pigments simultaneously. The mineralized bone was stained by fast green (Fig. 3A). Histomorphometric analysis revealed that on the 14th day of fracture healing, there was no significant difference in the proportion of cartilaginous callus distribution between the MMF group and the control group. However, MMF exposure significantly reduced the cartilage area/periosteal callus area (Cg.Ar./Ps.Cl.Ar.%) on the day 28 postfracture (Fig. 3B). In addition, MMF exposure significantly increased the mineralized area/periosteal callus area (Md.Ar./Ps.Cl.Ar.%) on both the 14th and 28th day postfracture (Fig. 3C).

### Effects of MMF exposure on osteoblastogenesis and osteoclastgenesis in fractured mice

The osteoblasts on the surface of the trabecular bone in the callus are shown in Fig. 3D. The statistical analysis demonstrated that MMF exposure increased the osteoblast number per bone surface (N.Ob/BS) of bone trabeculae on day 14 postfracture. However, there was no significant difference between the N.Ob/BS of control group and MMF group on day 28 (Fig. 3F, G).

The distribution of osteoclasts on the surface of the trabecular bone in the callus was stained by TRAP and shown in Fig. 3E. Statistical analysis revealed that there were no significant changes of osteoclast number per bone surface (N.Oc/BS), and osteoclast surface per bone surface (Oc.S/BS) in the callus of the control group and MMF group on day 14 postfracture. However, MMF exposure significantly reduced the N.Oc/BS and Oc.S/BS in callus on day 28 postfracture in callus (Fig. 3H, I).

**Fig. 4:**
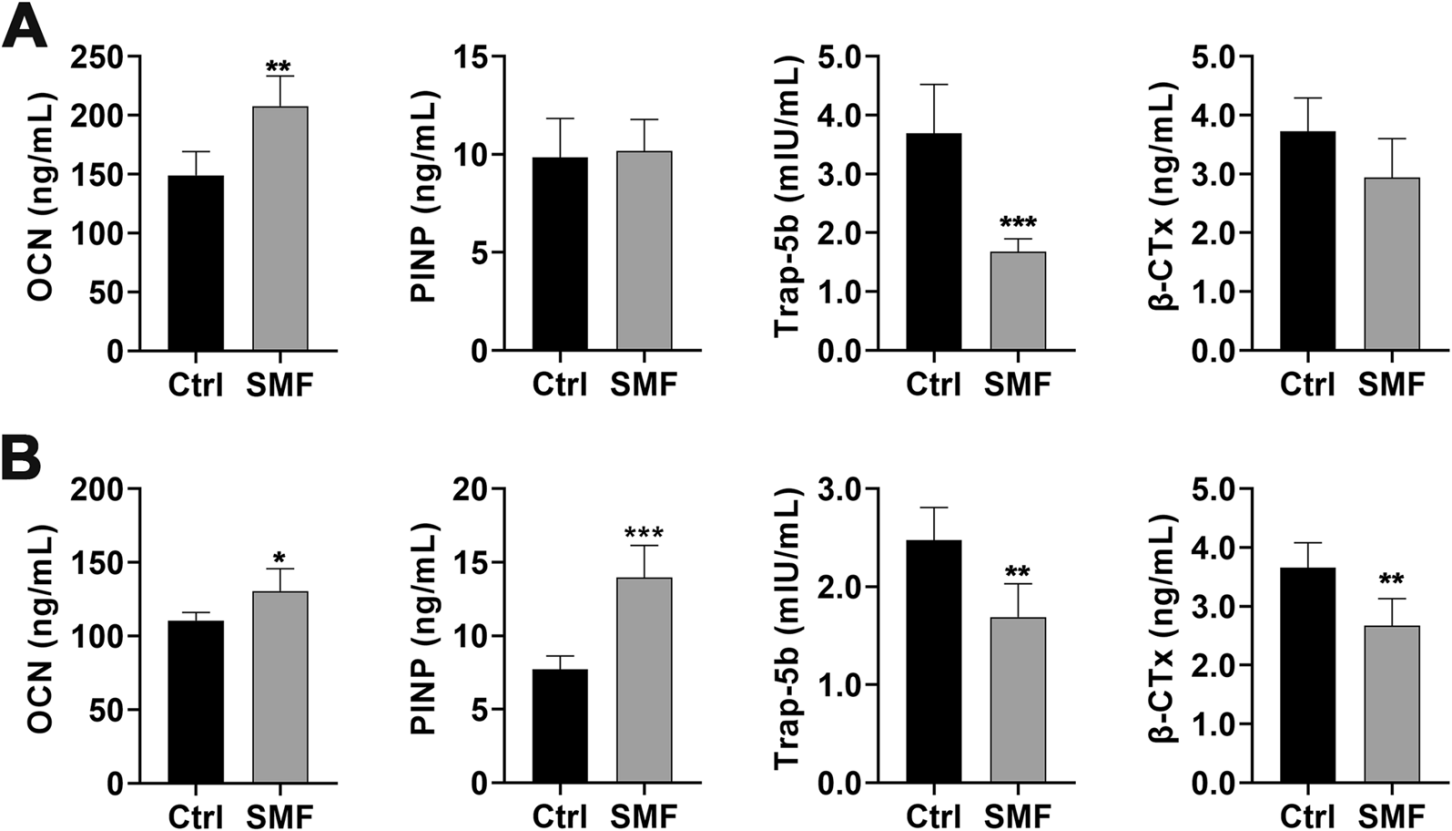
Effects of MMF exposure on the bone metabolism markers in serum of during fracture healing. **A** Effect of MMF exposure on the concentration of bone formation markers (OCN and PINP) and bone resorption markers (Trap-5b and β-CTx) in serum on day 14 postfracture. **B** Effect of MMF exposure on the concentration of OCN, PINP, Trap-5b and β-CTx in serum on day 28 postfracture. *n* = 8. Data represent the mean ± SD. **p < 0.01, ***p < 0.001

The bone formation makers OCN and PINP, and bone resorption makers TRAP-5b and β-CTX were used to evaluate bone turnover level in serum. The results showed that MMF exposure increased the concentration of OCN in serum while decreased the concentration of TRAP-5b in serum on day 14 postfracture (Fig. 4A). Meanwhile, MMF exposure increased the OCN and PINP concentration in serum, and decreased the TRAP-5b and β-CTX concentration on the 28th day of fracture healing (Fig. 4B).

### Effects of MMF exposure on iron content and distribution in fractured mice

**Fig. 5:**
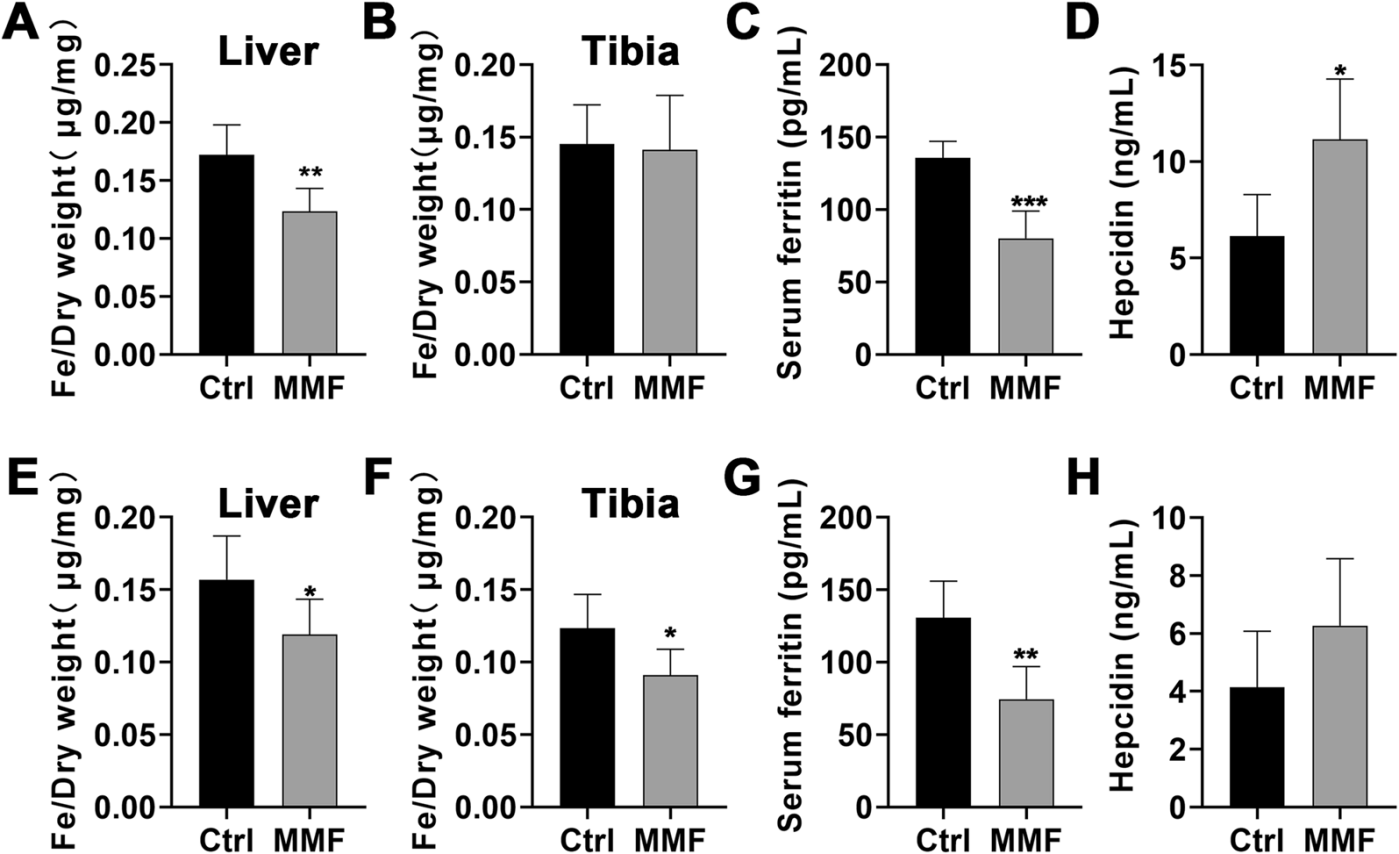
Effects of MMF exposure on iron metabolism during fracture healing. **A**–**D** Effect of MMF exposure on the total iron content in the liver (**A**) and fractured tibia (**B**), the serum ferritin (**C**), and hepcidin content in serum (**D**) on day 14 postfracture. **E**–**H** Effect of MMF exposure on the total iron content in the liver (**E**) and fractured tibia (**F**), the serum ferritin (**G**), and hepcidin content in serum (**H**) on day 28 postfracture. *n* = 8. Data represent the mean ± SD. **p < 0.01, ***p < 0.001

The status of iron metabolism is closely related to the growth and development of bones. The results showed that MMF exposure decreased iron content in the liver and serum ferritin on day 14 and day 28 postfracture (Fig. 5A, C, E, G). Meanwhile, MMF exposure decreased the iron content in fractured tibia on the day 28 of fracture healing (Fig. 5F). However, there was no significant difference in the iron content within the fractured tibia between the control group and the MMF group on the 14th day postfracture (Fig. 5B). Furthermore, MMF exposure increased the serum hepcidin content on day 14 postfracture, but had no significant impact on the serum hepcidin content on day 14 postfracture (Fig. 5D, H).

## Discussion

Several studies have previously documented that static magnetic fields (SMFs) with distinct parameters can exert varying effects on bone tissue and cells [11]. In this study, MMF exposure accelerated restoration of microstructure, enhanced mechanical properties, and improved morphology during the process of fracture healing. Meanwhile, MMF exposure modulated the distribution of osteoblasts and osteoclast in callus and had a significant impact the levels the bone turnover markers in serum. Moreover, MMF exposure exerted a regulatory influence on the iron content in callus and systemic iron metabolism during the fracture healing.

Routine blood parameters can serve as indicators of the fundamental physiological status of an organism, including the presence of inflammation [22]. It was observed that MMF exposure did not exert a significant effect on the levels of RBC, HGB, MCH, and PLT during fracture healing.

Inflammation are normal reactions in the early stages of fracture healing, and involved in promoting angiogenesis and initiating the repair process [23]. In contrast, dysregulated chronic inflammation is detrimental to fracture healing [24, 25]. The WBC, an important indicator of inflammation, was decreased on day 14 post-fracture in MMF exposure group mice. It is suggested that MMF exposure alleviated the inflammatory response during fracture healing. Previous studies have indicated the presence of two distinct waves of immune cells in fracture healing [26]. The first wave occurs during the early inflammatory phase and the second occurs during the late repair phase. The innate immune system is active in the inflammatory healing of hematoma. On the other side, lymphocytes, including B and T cells, are active in the healing callus during the late repair phase [26, 27]. Therefore, the effects of MMF on fracture healing and its immune regulation are worthy to research furtherly.

During the healing of fractures, there are a continuous improvement in the microstructures of the newly formed mineralized tissue in callus. In this study, MMF exposure significantly improved BMD and BV/TV, and accelerated the deposition of mineralized bone matrix in callus during fracture healing. As the fracture healing process advances to its final stages, there is a gradual return of mechanical properties along with an improved maturation and increased structural organization [28]. In this study, the results showed that the MMF exposure significantly improved structural parameters and material properties. Interestingly, the extent of improvement in mechanical properties, including the maximum load, surpassed the changes observed in microstructural features. It is suggested that MMF exposure caused the composition and structural orientation changes in newly formed bone tissue during fracture healing. Therefore, the composition ratio of collagen to minerals and the degree of structural alignment within the newly formed bone matrix under the influence of MMFs warrant further investigation.

The bone metabolism during fracture healing can be categorized into two major biological stages: endochondral ossification and coupled remodeling [4]. SMFs exposure has been shown to promote the chondrogenic and osteogenic differentiation of mesenchymal stem cells (MSCs) [29–33]. Endochondral ossification involves the cartilage formation and the transition from cartilage callus to hard callus [34].

In this study, the MMF exposure increased the mineralized area of the callus on the 14th and 28th day of fracture healing, accelerated the transition from cartilage callus to hard callus. Furthermore, the MMF exposure increased the number of osteoblasts in callus after 14 days of fracture healing. It is suggested that MMF exposure promoted fracture healing by accelerating the process of endochondral osteogenesis. During the stage of coupled remodeling, bone matrix is continually renewed through osteoblast-mediated bone formation and osteoclast-mediated bone resorption [35, 36]. In this study, there was no significant change on N.Ob/BS under MMF exposure, yet the concentrations of bone formation markers OCN and PINP were increased on day 28 postfracture. It is suggested that MMF exposure could improving the bone matrix formation ability of osteoblasts.

Osteoclast could participate in multiple stages of fracture healing, and indispensable for the coupled remodeling in the final stage of fracture healing [36]. Meanwhile, osteoclast-mediated bone resorption may exert a detrimental effect on the formation of mineralized bone matrix. In this study, the levels of TRAP-5b, β-CTx in serum, as well as the N.Oc/BS and Oc.S/BS in callus, were reduced after 28 days of MMF exposure. It is suggested that the MMF exposure inhibited the bone resorption on the stage of coupled remodeling during fracture healing. Studies demonstrated that the inflammation level in the microenvironment could affect the differentiation and bone resorption of osteoclasts [37]. Therefore, further investigation was warranted to explore the connection between the impact of MMF exposure on bone resorption and its regulation on inflammation during fracture healing.

Iron metabolism is closely related to bone health [38]. SMFs could influence bone homeostasis by regulating iron metabolism in various bone cells, including osteoblasts and osteoclasts [20, 39]. Iron overload could enhance bone resorption and inhibit bone formation, and iron-chelating agents have demonstrated their potential to alleviate this condition [40]. In this study, the iron content in liver and serum ferritin have been significantly decreased by MMF exposure on day 14 and day 28 postfracture. Meanwhile, the iron content in the fractured tibia of MMF exposure group was significantly decreased on day 28 postfracture. Hepcidin plays a central role in regulating the body's iron metabolism and is primarily expressed by the liver [41]. Research has demonstrated that an abnormal reduction in hepcidin levels can result in systemic iron accumulation, potentially leading to the development of osteoporosis [42]. In this study, the MMF exposure increased the content of hepcidin in serum on day 14 postfracture. These findings suggested that the promotion of fracture healing by MMF exposure was linked to its regulation of iron metabolism.

Changes in systemic iron metabolism could lead to symptoms, such as anemia [43]. Furthermore, fractures could result in significant blood loss and subsequent anemia in patients, which might lead to various health issues [44]. However, in this study, MMF exposure had no significant impact on HGB and MCH, indicating that it did not exacerbate anemia symptoms caused by fractures.

Previous studies found that MMF had no significant effect on the microstructure and mechanical properties of bones in normal mice while could suppressed bone loss in ovariectomized mice and HLU mice [15, 16, 45]. In this study, the MMF had increased bone formation and inhibited bone resorption in bone callus during fracture healing. These results suggested that the MMF have the potential for application in the treatment of orthopedic diseases characterized by the non-equilibrium bone metabolism, including fractures and osteoporosis.

In summary, this study indicated that MMF could promote fracture healing in mice by regulating the processes of endochondral osteogenesis and bone remodeling. These finding offers valuable insights into the biological mechanism of MMF exposure influences on fracture healing and provides a theoretical basis for its application in clinical bone fractures therapy.

## Materials and methods

### Animals and treatments

The 8-week-old male C57BL/6 mice were purchased from Beijing Vital River Laboratory Animal Technology Co., Ltd. and utilized to construct the model of open tibial fracture. The fracture surgeries were performed by specialized laboratory personnel with over 2 years of experience. The mice were anesthetized with isoflurane, then cut the skin with a scalpel, and the proximal tibia was drilled a hole with a 27 G needle. Cutting the diaphysis of the tibia to create a fracture, and then fix the fracture site through the hole with a stainless steel insect pin, as the tibia stabilized fractures. Then, the fractured mice were divided into four equal groups (*n* = 8), two groups of mice were exposed to a MMF, and the rest of the mice were exposed to geomagnetic field as control groups. Day 14 and 28 postfracture, the mice were culled by cervical dislocation under anesthesia. Blood samples were collected and stored at 4 °C for subsequent analyses. The collected tibia was preserved in 4% paraformaldehyde or at – 80 °C for subsequent analyses, respectively. All animal protocols used in this study were approved by the Lab Animal Ethics and Welfare Committee of Northwestern Polytechnical University.

### Routine blood and serum biochemical assay

The blood samples were mixed with EDTA-K2 anticoagulant immediately after collection, and routine blood analysis was conducted using a Sysmex xs-800i automated hematology analyzer (Sysmex TMC, KOBE, Japan).

The blood samples were collected and kept standing still at 4 ℃ until the serum separated out before being centrifuged at 10,000*g* for 10 min, after which time the supernatant was collected to obtain the serum. Serum markers for bone metabolism, including propeptide of type I procollagen (PINP), osteocalcin (OCN), beta-isomer of the C-terminal telopeptide of type I collagen (β-CTX), and tartrate-resistant acid phosphatase 5 b (TRAP-5b) were examined by respective mouse enzyme linked immunosorbent assay (ELISA) kits (JiangLai biological, Shanghai, China). Meanwhile, the serum ferritin and hepcidin content of mice was detected using an ELISA kit (JiangLai biological, Shanghai, China). All procedures were strictly performed in line with the instructions of the manufacturers.

### Micro-CT

The fractured tibia was collected and the intramedullary pins as well as the external soft tissues were carefully removed. The post fracture tibia at different healing timepoints were scanned using Micro-computed tomography (Micro-CT) (VivaCT80, SCANCO Medical AG, Bassersdorf, Switzerland). Scanning was performed at 70 kV, 114 μA, 250 ms without a filter. The parameters of the fracture site were three-dimensionally reconstructed using NRecon software (Bruker, BILLERICA, Massachusetts, USA). Carried out on the middle 2 mm slices (1 mm above and below the fracture line) as the region of fracture callus, and low-density tissue was distinguished from high-density tissue on the basis of different thresholds. The micro structure parameters of fracture area were analyzed using CTAn software (Bruker, BILLERICA, Massachusetts, USA). The following measures of fracture callus structure and composition were evaluated for each specimen: callus bone mineral density (BMD), callus mineralized volume fraction (BV/TV, %), trabecular thickness (Tb.Th), trabecular number (Tb.N), and trabecular separation (Tb.Sp) were analyzed.

### Biomechanical examination

The bone mechanical properties on day 28 postfracture was evaluated by three-point bending test using the Instron-5943 Universal Material Testing Machine (Instron 5943; Instron, Canton, MA, USA). The span of two supports was 8 mm and loading rate was 1 mm/min until the callus fracture again. The load–displacement curve was obtained from the Instron software and used for calculate the parameters of structural properties, including ultimate load, ultimate displacement, stiffness, and bending energy absorption. The stress–strain curve that was normalized by the geometrical measurements was obtained from the MATLAB software (The MathWorks, Inc., Natick, MA, USA), which were used for calculate the parameters of material properties, including ultimate stress, ultimate strain, elastic modulus, and toughness.

### Histochemical examination

Histochemical examination of the fracture callus was performed on the 14th and 28th days after the fracture. The fractured tibia was fixed in 4% paraformaldehyde for 48 h, and decalcified by 10% EDTA. Then, the fractured tibia was embedded in paraffin and sectioned in 5 µm thick slices. Safranin orange/fast green (SFO/FG) staining was used to detect the distribution of cartilage and mineralized area in callus, cartilage tissue was stained in red, and mineralized bone tissue was stained in green. Alcian blue staining was also used for cartilage staining at fracture callus sites.

HE staining was used to detect the basic morphology of fracture callus and to count osteoblasts, and the osteoblasts adhere to the surface of bone trabeculae in a long spindle shape. Tartrate-resistant acid phosphatase staining (TRAP; Sigma-Aldrich, Burlington, MA, USA) staining was used to evaluate osteoclast in fracture callus, the osteoclasts are stained red. The osteoblast number per bone surface (N.Ob/BS), osteoclast number per bone surface (N.Oc/BS), and osteoclast surface per bone surface (Oc.S/BS) have been analyzed via Fluorescence microscope and Image J software (NIH, Bethesda, MD, USA)following established procedures by personnel with over 5 years of experience in organizational assessment [20].

### Determination of Fe content in tissue

The Fe content in the liver and fractured tibia was determined by atomic absorption spectrometry (AAS; Analytik Jena, Jena, Germany). The liver and fractured tibia were collected from the sacrificed mice after the cardiac perfusion by normal saline solution. Dried the tissues under 120 ℃ for 6 h, and measured its dry weight. After that, heated the tissues at 600 ℃ for 6 h to ash the tissue, then dissolved in 1 ml 65% HNO3. Dilute the samples with ddH2O to a suitable concentration, then the Fe concentration was measured by flame AAS. The Fe content in tissues was calculated as the mass fraction between the mass of the Fe and tissue dry weight, respectively.

### Statistical analysis

All statistical analyses were performed using GraphPad Prism statistical software for Windows (version 8, GraphPad Software Inc., San Diego, CA, USA). The data are expressed as the mean ± standard deviation. The normal distribution was tested by Shapiro–Wilk normality test with *P* > 0.05. Two-tailed Student *t* tests was used to compare the differences between two groups, and one-way ANOVA was used to analyze the differences between multiple treatment groups. For all statistical tests, *P* < 0.05 was considered to indicate statistical differences.

## Data Availability

Not applicable.

## Abbreviations

SMF: Static magnetic fields
MMF: Moderate static magnetic field
GMF: Geomagnetic field
Micro-CT: Micro-computed tomography
BMD: Bone mineral density
BV/TV: Bone volume per tissue volume
Tb.Th: Trabecular thickness
Tb.N: Trabecular number
Tb.Sp: Trabecular separation
HyMF: Hypomagnetic field
HiSMF: High static magnetic field
WBC: White blood cell count
LYMPH: Lymphocyte
RBC: Red blood cell count
HGB: Hemoglobin
MCH: Mean corpuscular hemoglobin
PLT: Platelet count
Cg.Ar./Ps.Cl.Ar.%: Cartilage area/periosteal callus area
Md.Ar./Ps.Cl.Ar.%: Mineralized area/periosteal callus area
PINP: Propeptide of type I procollagen
OCN: Osteocalcin
β-CTX: Beta-isomer of the C-terminal telopeptide of type I collagen
TRAP-5b: Tartrate-resistant acid phosphatase 5 b
MSCs: Mesenchymal stem cells
